# (Poly)phenols: Mechanisms of action and efficacy of contemporary supplements for exercise recovery and performance

**DOI:** 10.1113/EP093724

**Published:** 2026-06-12

**Authors:** Glyn Howatson, Tom Clifford

**Affiliations:** ^1^ Department of Sport Exercise and Rehabilitation Northumbria University Newcastle‑upon‑Tyne UK; ^2^ Water Research Group North West University Potchefstroom South Africa; ^3^ School of Sport, Exercise and Health Sciences Loughborough University Loughborough UK

**Keywords:** athletic performance, exercise‐induced muscle damage, fatigue, recovery

## Abstract

(Poly)phenols are a diverse group of bioactive chemical compounds present in a wide range of plant‐ and animal‐based foods. Several thousands of (poly)phenols exist; many have potent biological effects, most notably antioxidant, anti‐inflammatory and vasodilatory. As such, they are of growing interest as nutritional interventions for health maintenance and disease prevention, but also for athletes aiming to enhance recovery and performance. Although most of their recovery and performance benefits are ascribed to their antioxidant actions, they are unlikely to function as antioxidants in vivo. Rather, they might upregulate the transcription factor nuclear factor‐erythroid‐2‐related factor (Nrf2), which, by upregulating various endogenous antioxidants, could modulate cellular redox balance. Likewise, their anti‐inflammatory functions are likely to be driven by their effects on the transcription factor nuclear factor kappa B, which regulates numerous pro‐inflammatory genes. Although receiving far less attention, (poly)phenols might also increase mitochondrial biogenesis and fat oxidation, both of which could enhance endurance exercise performance. These biological effects have consistently been demonstrated in vitro, but there is less mechanistic support for them in human studies, despite several clinical trials reporting, albeit inconsistently, improved recovery and exercise performance. Translating the findings from in vitro and animal models to humans is challenging, in large part owing to the poor bioavailability of most (poly)phenols and thus the limited amount of the intact metabolites available for biological effects. This brief review outlines the primary mechanisms through which (poly)phenols might influence exercise recovery and performance and highlights some of the evidence from research that investigates popular (poly)phenol supplements on key performance and recovery outcomes.

## Abstract (highlights)

- **What is the topic of this review?**
  This review examines the influence of dietary (poly)phenols on the physiology of exercise; specifically, summarising the proposed mechanisms that underpin recovery and athletic performance.
- **What advances does it highlight?**
  The review highlights the emerging evidence exploring the potential mechanisms of (poly)phenols in human performance. We summarise the biological effects of (poly)phenols from in vitro to human studies and provide a current viewpoint on their efficacy using contemporary examples of (poly)phenols that have been explored for their effects on exercise recovery and performance.

## 1 INTRODUCTION AND BACKGROUND

Phytochemicals, compounds found in plants, have received great interest because of the potential effects on improving health and reducing disease risk (Bell et al., 2014). Of particular interest are those classified as phenols and polyphenols, collectively termed (poly)phenols (Frank et al., 2020). They are chemical compounds, mostly secondary metabolites found in plants, that have a range of important botanical functions, including growth, pigmentation and protection from ultraviolet stress (Zagoskina et al., 2023). The several thousands of (poly)phenols known to exist are commonly classified into four categories, based on their chemical structure: flavonoids, stilbenes, lignans and phenolic acids (Ciupei et al., 2024). These (poly)phenols are present in leaves, herbs, fruits and vegetables; examples of abundant sources might include tea, coffee, wine, grapes, cocoa, berries, cherries, blackcurrants, pomegranates, and particularly brightly coloured red and blue fruits (Bowtell & Kelly, 2019; Zagoskina et al., 2023). Detailed definitions of (poly)phenols are provided elsewhere (Frank et al., 2020), but for added clarity many of the aforementioned fruits and vegetables contain the various classifications of (poly)phenols, phenolics acids, but also parent anthocyanins (e.g., cyanidin, peonidin, delphinidin and malvidin) that are metabolised quickly after consumption to various bioactive phenolic compounds. Although these compounds are collectively known for their antioxidant properties, and therefore effects on cellular redox balance, it is now recognised that many dietary (poly)phenols are pleiotropic compounds that exert a wide range of biological activities that include anti‐inflammatory, anti‐atherogenic, anti‐carcinogenic, anti‐microbial, vasodilatory and chemoprotective effects (Pandey & Rizvi, 2009). Given their diverse functionality and widespread availability, it is unsurprising that interest in the potential health benefits of (poly)phenols continues to expand. Indeed, in 2022 the first ever dietary guidelines for recommended intakes of polyphenols (flavon‐3‐ols) for cardiometabolic health were published (Pandey & Rizvi, 2009), which is supported by dietary bioactive guidelines (Crowe‐White et al., 2022). There is also strong interest in the potential benefits of (poly)phenols in sport and exercise, with several studies examining their effects on human performance and recovery from strenuous exercise. This brief review examines the potential mechanisms that might underpin the recovery and performance‐enhancing effects of (poly)phenol‐rich foods and provides a précis of some underlying exemplar evidence from widely studied and popular (poly)phenol interventions.

## 2 POTENTIAL MECHANISMS OF ACTION OF (POLY)PHENOLS FOR PERFORMANCE AND RECOVERY

### 2.1 Antioxidant

**FIGURE 1:**
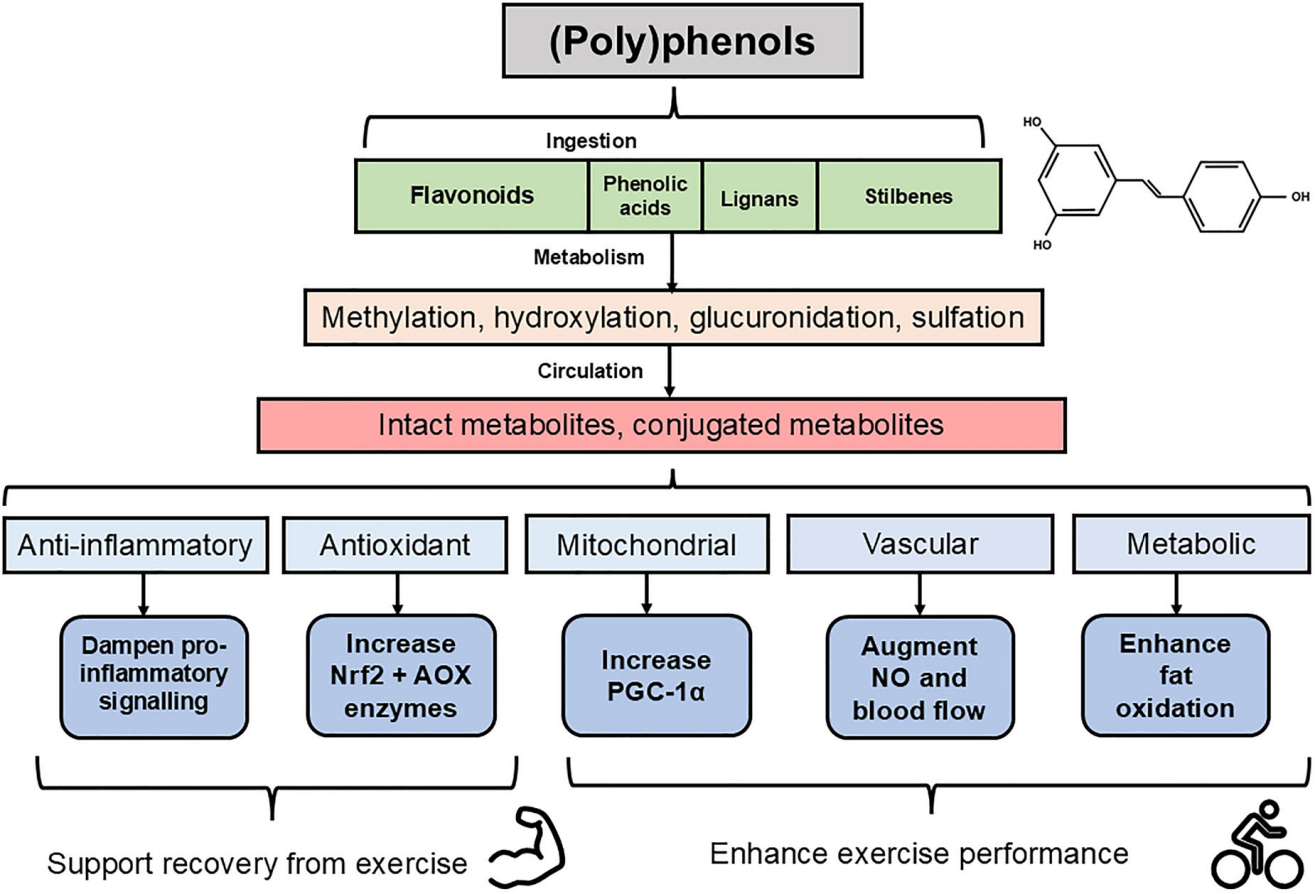
In brief, a schematic diagram of (poly)phenol metabolism and putative physiological effects. (Poly)phenols go through first‐pass metabolism in the stomach before many are extensively metabolised in the liver, intestines and colon. (Poly)phenols are subject to methylation, sulfation and glucuronidation reactions, meaning that many of the metabolites reaching the circulation are in their conjugated forms. Those (poly)phenols (intact or conjugated) that do reach the circulation might have a variety of physiological effects that could impact exercise recovery and performance. Abbreviations: AOX, antioxidant; NO, nitric oxide; Nrf2, nuclear factor‐erythroid‐2‐related factor; PGC1‐α, peroxisome proliferator‐activated receptor gamma coactivator‐1α.

(Poly)phenols can have both antioxidant and pro‐oxidant actions in vitro (Halliwell, 2008), but it is the antioxidant actions that have received the most attention. Historically, antioxidant effects have been considered the major mechanism by which these (poly)phenols might modulate exercise performance and attenuate markers of exercise‐induced muscle damage (EIMD; Margaritelis et al., 2020). Evidence of direct antioxidant actions in vivo is lacking, largely owing to the difficulty in measuring reactive oxygen species (ROS; Cobley et al., 2024), but there is considerable evidence that biomarkers of oxidative stress, such as lipid, DNA or protein oxidation, are reduced in humans with various (poly)phenols (Gonzalez‐Gomez et al., 2025; Sarkhosh‐Khorasani et al., 2021). Notwithstanding, it is unclear how or whether (poly)phenols maintain their antioxidant potential after digestion, given that many (poly)phenols are extensively metabolised in the liver, colon and intestines, which might disable their antioxidant function (Augustin et al., 2008; Halliwell, 2008); see Figure 1. It is possible, however, that the bio‐transformed, conjugated metabolites possess antioxidant functions, but not at the same potency (Wojtunik‐Kulesza et al., 2020). In fact, the structural transformation of some (poly)phenols post‐ingestion could give them pro‐oxidant functions (Halliwell, 2008). Interestingly, recent research in the field of sensory nutrition suggested that astringent‐tasting (poly)phenols, such as epicatechins and procyanidins, might produce ROS (superoxide; O_2_^•−^) in the gastrointestinal (GI) tract, which, in turn, activates the sympathetic nervous system and modulates blood flow; see section 2.2 ‘*Muscle perfusion*’ below for further details (Fushimi et al., 2023; Osakabe et al., 2025). It is also possible that (poly)phenols are direct antioxidants in the stomach or small intestine prior to metabolism, which could help to maintain the structure of the gastrointestinal barrier during high‐intensity exercise (Szymanski et al., 2018). However, it is unclear whether this would markedly improve athletic performance or attenuate markers of EIMD.

Some of the intact (poly)phenols can reach the circulation, but the bioavailability of these compounds (often measured in blood or urine) is often low (Squires, Walshe, Cheung et al., 2024), with sometimes <1% of the intact compound detected (Scalbert & Williamson, 2000). For example, in a recent study a ∼200 mg dose of curcumin resulted in nanomolar concentrations of total curcuminoids reaching the plasma in the ∼2 h post‐ingestion (Thorley et al., 2024). Peak concentrations are typically reached a few hours after intake and are markedly lower than that of the endogenous antioxidants, such as glutathione and uric acid; as such, they are unlikely to reach the circulation in sufficient quantities to scavenge ROS directly at the target cells (Forman et al., 2014). Hence, the contemporary view is that (poly)phenols probably exert their antioxidant effects indirectly, at least outside the gut, by inducing redox and other antioxidant enzymes that might have an impact on cellular redox balance and oxidative stress (Forman et al., 2014; Halliwell, 2008). Indeed, there is evidence that (poly)phenols induce redox enzymes, such as superoxide dismutase, catalase, thioredoxin and glutathione peroxidase, through activation of the transcription factor nuclear factor‐erythroid‐2‐related factor 2 (Nrf2; Zhou et al., 2019). In addition, the recent work suggesting that (poly)phenols might exert physiological effects via sensory nerves in the GI tract is another mechanism by which they might evoke beneficial health effects despite their poor bioavailability (Koizumi et al., 2021).

The regulation of canonical and non‐canonical Nrf2 activation has been described in detail elsewhere (Zhang, 2025). In brief, both pathways involve the protein kelch‐like ECH‐associated protein 1 (Keap‐1). In basal conditions, Keap‐1 retains Nrf2 in the cytoplasm, preventing its activity. However, given that many (poly)phenols have electrophilic properties, they can react with the thiol groups of cysteine residues on Keap‐1 (canonical pathway) or phosphorylate Nrf2, either of which frees Nrf2 for translocation to the nucleus, where it induces expression of several redox and inflammatory‐related genes (Zhou et al., 2019). The electrophilic activity of (poly)phenols suggests that the ‘antioxidant’ actions of (poly)phenols might, counter‐intuitively, arise from an initial pro‐oxidant stimulus that triggers antioxidant defences. In theory, an Nrf2‐induced decrease in ROS could attenuate muscle fatigue during high‐intensity exercise or help to restore cellular redox balance after muscle‐damaging exercise, limiting oxidative stress that might affect neuromuscular function in the days after exercise.

To date, most of the evidence to support effects of (poly)phenols on the Nrf2 pathway has been in cell culture. In a recent systematic review of human studies, limited and inconsistent evidence of increased Nrf2 activity after phytochemical supplementation was evident (Clifford et al., 2021); however, more recent exercise‐based studies have reported some encouraging findings. A small study (Thorley et al., 2024) showed that although neither curcumin nor exercise was able to increase Nrf2 activity markedly in peripheral blood mononuclear cells, regression analysis suggested that Nrf2 activity was higher in those with greater plasma curcuminoid concentrations. In a recent *ex vivo* study, the phytochemical sulforaphane (found in broccoli sprouts) applied to peripheral blood mononuclear cells collected postexercise was able to augment Nrf2 activity (Rodriguez et al., 2025). In addition, a recent study with the alga *Tetraselmis chuii*, which contains, amongst other bioactives, (poly)phenols, increased the expression of Nrf2 (∼1.6‐fold) and other key antioxidant enzymes in skeletal muscle, in addition to peak oxygen uptake, when consumed for 14 days (Cocksedge et al., 2025). These human studies are promising, but it is important to note that our progress in understanding how these compounds impact Nrf2 in humans is significantly hampered by the difficulty in measuring Nrf2 in vivo, given that it has a very short half‐life (Cuadrado et al., 2019). As such, it is more common for studies to measure downstream targets that are more stable, such as NAD(P)H:quinone oxidoreductase 1 (NQO1) and heme oxygenase 1 (HMOX1), to provide evidence of Nrf2 activation (Yagishita et al., 2020). Ideally, future studies will measure Nrf2 alongside an array of downstream targets to enhance our understanding of how (poly)phenols and other phytochemicals might impact this pathway.

### 2.2 Muscle perfusion

Another key mechanism by which (poly)phenols might enhance exercise performance is by augmenting muscle blood flow, hence the contribution of oxidative derived (aerobic) energy production during exercise (Bowtell & Kelly, 2019; Keane et al., 2018). Indeed, many (poly)phenols are thought to encourage endothelium‐dependent vasodilatation by increasing nitric oxide (NO) bioactivity via several mechanisms, including reducing the formation of ROS that react with NO, increasing intracellular Ca^2+^ concentrations, inhibiting nuclear factor kappa B (NF‐κB) signalling (see section 2.5 Inflammation), and activating the serine/threonine kinase Akt and endothelial nitric oxide pathway (Serreli & Deiana, 2023). Recent work has shown that NO and blood flow are at least partly augmented via the actions of astringent (poly)phenols on transient receptor potential channels in the GI tract, which increase sympathetic nervous system activity (Koizumi et al., 2021). These effects may be mediated, in part, by the pro‐oxidant or hormetic effects of some (poly)phenols; that is, low levels of ROS or what might be termed astringent stress might stimulate beneficial cellular adaptations (Fushimi et al., 2023; Osakabe et al., 2024). A study in rats showed that the astringent procyanidin (poly)phenol increased blood flow, an effect that could be blunted by administration of the free radical scavenger *N*‐acetyl‐l‐cysteine or by transient receptor potential channel blockers, which dampen sympathetic nervous system activity (Fushimi et al., 2023). Although several in vitro studies and animal studies have shown that different (poly)phenols can increase NO and vasodilatation via these mechanisms (Osakabe et al., 2024; Stoclet et al., 2004), there remain limited data on these effects in humans, especially direct increases in NO bioactivity. Nonetheless, several clinical trials have shown that (poly)phenol supplementation in the form of blueberries can increase blood flow, as measured by flow‐mediated dilatation in the brachial artery (Wood et al., 2023). Flow‐mediated dilatation cannot be used during exercise, but some studies have used near‐infrared spectroscopy to estimate local muscle perfusion (Keane et al., 2018; Morgan et al., 2019). Indeed, studies with anthocyanin‐rich tart cherries have shown that supplementation can positively influence some indices of local oxygenation in the vastus lateralis during exercise. Further research is needed to clarify the precise mechanisms, especially the role of NO, but an increase in muscle perfusion during exercise is a plausible explanation for how (poly)phenols could enhance exercise performance.

### 2.3 Mitochondrial biogenesis

Although far less explored than their antioxidant or vascular effects, (poly)phenols might also positively influence mitochondrial biogenesis. Although such changes would not be expected to impact EIMD, longer‐term intake might enhance endurance exercise performance. Regarding mitochondrial biogenesis, several animal studies have shown that (poly)phenols can stimulate peroxisome proliferator‐activated receptor gamma coactivator‐1α (PGC‐1α; Lagouge et al., 2006), which is a key transcriptional activator of mitochondrial biogenesis and function. In one study in mice, quercetin intake increased endurance performance alongside increasing gene expression of PGC‐1α, and other markers of mitochondrial biogenesis, such as sirtuin 1 (SIRT1) and cytochrome *c* (Davis et al., 2009). There is also some evidence of (poly)phenols increasing mitochondrial biogenesis in human skeletal muscle; a study in type 2 diabetes patients found that consuming epicatechin‐rich cocoa flavanols for 3 months increased SIRT1, PGC1‐α and the mitochondrial proteins mitofilin and prolin (Taub et al., 2012).

### 2.4 Fat oxidation

During longer‐duration higher‐intensity endurance exercise (>90–120 min), during which there is a heavy reliance on the finite muscle glycogen stores for energy, an increased capacity to oxidise the almost limitless fat stores and reduce the reliance on glycogen might delay the onset of fatigue and enhance performance (Hawley, 2002). Some (poly)phenols have been shown to increase the oxidation of fats during exercise, although the effects are inconsistent. For example, human studies have shown that anthocyanin‐rich New Zealand blackcurrants can increase fat oxidation during submaximal cycling exercise (Cook et al., 2026). A recent study suggested that these effects might be at least partly mediated by greater intramuscular triglyceride use in type I muscle fibres during prolonged exercise (Jones et al., 2025). However, New Zealand blackcurrants did not impact the rate of glycogen utilisation or the exercise capacity in this study. In contrast, the (poly)phenol epigallocatechin gallate was shown to decrease fat oxidation rate by 32% during a graded high‐intensity cycling test (Churm et al., 2023). The underlying mechanisms for how (poly)phenols impact fat oxidation have not been well examined, but it has been suggested that they might target the sympathetic nervous system or increase the activity or expression of genes involved in lipid metabolism (Hodgson et al., 2013), such as hormone‐sensitive lipase. However, there are currently limited data in humans to support these effects (Jones et al., 2025; Strauss et al., 2018).

### 2.5 Inflammation

One of the main mechanisms by which (poly)phenols are thought to modify markers of EIMD is via their anti‐inflammatory effects. Inflammation is a complex process, and there are many different pathways that (poly)phenols have been proposed to modulate, including the cyclooxygenase (COX), lipoxygenase (LOX) and NF‐κB signalling pathways, in addition to various mitogen‐activated protein kinases (MAPKs; Yahfoufi et al., 2018). By inhibiting the activity of COX and LOX enzymes, (poly)phenols might attenuate the production of, amongst other mediators, prostaglandins, thromboxanes and leukotrienes, which play key roles in inflammation (Mitjavila & Moreno, 2012; Yahfoufi et al., 2018). Given that cyclooxygenase 2 (COX‐2) is the enzyme that most non‐steroidal anti‐inflammatory drugs are designed to inhibit, (poly)phenols could share similarities to non‐steroidal anti‐inflammatory drugs, although this is yet to be examined robustly in human studies. The major pathway that many (poly)phenols have been shown to modulate, at least in vitro and in animal models, is the NF‐κB pathway, which regulates the expression of several key inflammatory genes, including cytokines, chemokines, growth factors and COX‐2 (Vendrame & Klimis‐Zacas, 2015). (Poly)phenols and their derivatives can inhibit the NF‐κB pathway by decreasing its nuclear translocation (Vendrame & Klimis‐Zacas, 2015), but also by activating Nrf2 and reducing oxidative stress and cytokine expression (Kobayashi et al., 2016; Zhang & Tsao, 2016), thereby providing a potential mechanistic pathway that underpins the decrease in inflammatory mediators (Hariri et al., 2024).

It is important to note that although there are several studies (summarised in a systematic review) suggesting that (poly)phenols in the form of various purified anthocyanins can reduce some markers of inflammation in humans, particularly plasma cytokine levels (Hariri et al., 2024), there are limited randomised controlled trials examining their effects on NF‐κB or COX‐2 expression. Of relevance to exercise, one study did find that consuming a (poly)phenol‐rich protein drink before and after muscle‐damaging exercise (300 lower‐limb eccentric muscle contractions) downregulated NF‐κB signalling in skeletal muscle, as evidenced by decreases in relevant genes, including interleukin receptor 1 receptor like 1, C‐X‐C motif chemokine ligand 1 and intracellular adhesion molecule 1 (Jameson et al., 2021). These changes were accompanied by a quicker return to baseline neuromuscular function in the 48 h postexercise, suggesting the decreased inflammatory response could have contributed to the accelerated recovery of muscle function.

In the following sections, we provide an overview of the potential for (poly)phenols to influence exercise recovery and performance in a positive manner, with a focus on human studies. The following sections are not intended to be an exhaustive review of all (poly)phenols, but instead a brief review of selected contemporary (poly)phenol‐rich foods in human performance and recovery: tart cherries, blackcurrants, pomegranate and curcumin. For more detailed reviews on a wider range of (poly)phenol interventions in exercise performance and recovery, the reader is directed elsewhere (Bowtell & Kelly, 2019; Mason et al., 2020).

### 2.6 Tart cherries

#### 2.6.1 Effects on exercise recovery

Tart cherries have been shown to facilitate recovery following different exercise types, such as heavy resistance training (eccentric contractions) in untrained and well‐trained people (Bowtell et al., 2011; Connolly et al., 2006); following long‐distance running (Howatson et al., 2010), after intermittent sprinting (Bell et al., 2016) and after cycling exercise (Bell et al., 2015), although these effects have not been observed universally (Abbott et al., 2020; Squires, Walshe, Dodd et al., 2024). The positive effects have largely been attributed to reduced oxidative stress and inflammation, despite relatively few studies measuring these parameters. Notwithstanding, some attempts have been made to examine indices of inflammation, but the results are generally inconsistent. Markers of oxidative stress have rarely been measured, probably owing to the complexity of the assays and the limitations of these measures in making inferences to redox balance. The first studies that measured oxidative stress (Bowtell et al., 2011; Howatson et al., 2010) showed that after a marathon run and eccentric biased resistance training, plasma lipid peroxidation (thiobarbituric acid) and protein carbonyls were reduced, respectively; both showing improved recovery of muscle function. These earlier studies had numerous limitations but are supported by a recent study that used heavy eccentric resistance exercise as a model to explore the mechanisms by which tart cherries might impact neuromuscular recovery (Wangdi et al., 2022). Their study showed that tart cherry concentrate and fresh pressed juice taken in the days before and after the damaging exercise upregulated skeletal muscle concentrations of key antioxidant enzymes (e.g., glutathione peroxidase) and was accompanied by improved recovery of muscle strength. This evidence directly supports the idea that (poly)phenols induce redox enzymes through activation of Nrf2 (Zhou et al., 2019) and warrants further exploration. On balance, tart cherries have been shown to have a positively influence on aspects of exercise recovery, although there is still a limited understanding of how they might exert their effects.

#### 2.6.2 Effect on exercise performance

The effect of tart cherries on exercise performance is less clear. A meta‐analysis showed a small positive effect on endurance performance when data from 10 studies were pooled (Gao & Chilibeck, 2020); however, only 20% of the included studies reported statistically significant changes, hence these results should be treated with caution. For example, Davis & Bellar (2019) showed no improvement in cycling time to exhaustion at 70% peak oxygen uptake or muscle oxygenation following 6 days (500 mg/day) of freeze‐dried tart cherry powder. The lack of change in time to exhaustion and muscle oxygenation during cycling was also supported by Keane et al. (2018), although their study, which used 30 mL of acutely ingested Montmorency tart cherry concentrate, did report some modest improvements in the latter part of a 60 s sprint performance. Conversely, others (Morgan et al., 2019) showed that performance and muscle oxygenation during a 16 km cycling time trial were improved after consuming tart cherry capsules for the preceding 6 days. More recently (Horiuchi et al., 2023) provided more convincing evidence of their ergogenic effects, exploring the effect of 4 days of supplementation with tart cherry capsules (1200 mg, containing 100 mg of anthocyanin) before exposure to an incremental and maximal exercise semi‐recumbent cycling challenge in hypoxia that equated to ∼3800 m altitude (fraction of inspired O_2_ = 0.13). In this cross‐over design, tart cherries (in comparison to a placebo control) improved the time to exhaustion during an incremental test, with a concomitant reduction in deoxyhaemoglobin, increased tissue oxygen saturation and a reduction in urinary 8‐hydro‐20 deoxyguanosine (an index of oxidative damage to DNA). Although the collective body of evidence is inconsistent, it seems that tart cherries might improve muscle perfusion with oxygen that can translate to an improvement in performance, particularly in environments where oxygen utilisation might be compromised.

### 2.7 Pomegranate

#### 2.7.1 Effects on exercise recovery

Pomegranates are rich in several (poly)phenols, especially ellagitannin, punicalagins and ellagic acids. Mostly in the form of pomegranate juice, several studies have examined the effects on markers of EIMD, which were recently summarised in a meta‐analysis (Belyani et al., 2025). Early findings showed promise (Trombold et al., 2010, 2011) and illustrated that consuming 500 mL of pomegranate juice in the days before and after muscle‐damaging exercise accelerated the recovery of isometric muscle strength. However, these findings are not consistent with more recent research in untrained men (Lamb et al., 2019) showing that a much smaller dose of 250 mL of pomegranate juice had no effect on indices of muscle damage compared with a placebo control, indicating that efficacy might be dose dependent. The recent meta‐analysis (Belyani et al., 2025), which included 10 studies, concluded that there is currently insufficient evidence to support the use of pomegranate interventions for most indices of EIMD.

#### 2.7.2 Effect on exercise performance

Acutely ingested pomegranate extract (1000 mg) has been shown to improve running performance (time to exhaustion at 90% and 100% of peak treadmill velocity attained during a peak oxygen uptake test) and vascular function (Trexler et al., 2014). However, these findings are not consistent across studies. Other work (Crum et al., 2017; Trinity et al., 2014) showed no effect on cycling performance (from 7 days of consumption of 500 mL of a commercially available juice) or repeated sprint and resistance training performance (Roelofs et al., 2017), although that study, which also used 1000 mg of acutely consumed pomegranate extract, did report improvements in blood vessel diameter 30 min post‐ingestion), which provides some support to previous research (Trexler et al., 2014). Furthermore, a study (Torregrosa‐Garcia et al., 2019) in which pomegranate extract was supplemented (750 mg/day) for 2 weeks reported improved time to exhaustion and submaximal exercise performance in trained cyclists, which was speculatively attributed, at least in part, to improved vascular function, despite no direct measures included. On balance, it seems that pomegranate interventions might improve vascular function and could therefore enhance the capacity for muscle perfusion and improve exercise performance.

### 2.8 Blackcurrant

#### 2.8.1 Effects on exercise recovery

Similar to tart cherries, blackcurrants are high in anthocyanins and anthocyanidins and therefore possess biological potential to support recovery from exercise. A recent narrative review (Willems et al., 2025) summarised a series of studies that examined the effects of (poly)phenol‐rich blackcurrant supplements on markers of exercise recovery. Of these studies, four were more focused on acute exercise responses (reduced blood lactate and endurance performance) and impact on subsequent performance, as opposed to recovery per se. In general, there were mixed results; some studies showed some positive effects on postexercise muscle soreness, in addition to reduced concentrations of plasma protein carbonyls and creatine kinase (Hunt et al., 2021; Lyall et al., 2009), but the recovery of neuromuscular function was unaffected after consumption of blackcurrant extract. Other studies also failed to find positive effects of blackcurrant supplements (Costello et al., 2020; Hutchison et al., 2016) on indices of muscle damage and recovery (muscle soreness and restoration of strength) following strenuous exercise. Consequently, the evidence to support the use of blackcurrant interventions for exercise recovery is weak, although more research is required before definitive conclusions can be drawn.

#### 2.8.2 Effect on exercise performance

In contrast to the studies on recovery, numerous studies have shown that blackcurrant supplements can enhance exercise performance (Willems et al., 2025), ostensibly by a combination of modulating blood flow (Cook & Willems, 2019), increasing fat oxidation (Hiles et al., 2020) and attenuating oxidative stress (Hurst et al., 2020). Initial work in this area, which used a 7‐day loading phase of 300 mg of blackcurrant extract (Cook et al., 2015), showed ∼2.4% improvement in 16.1 km time‐trial cycling performance when compared with a placebo. These observations were supported in a subsequent trial (Perkins et al., 2015), which showed that the same dosing strategy of blackcurrant extract improved the total distance covered during intermittent sprint treadmill running by a staggering 11%. Performance improvements have also been reported in other cycling tasks, intermittent running and sport climbing (Godwin et al., 2017; Murphy et al., 2017; Potter et al., 2020). More recent studies have produced mixed findings. Montanari et al. (2026) showed no effect of blackcurrants on an isometric fatiguing task of the quadriceps, despite using a very similar dose over a 7‐day period. Likewise, 4 km cycling time‐trial performance was not improved following acute and longer‐term (7 days) intake of blackcurrant extract (Morton et al., 2025). Accordingly, blackcurrant intake has a mixed evidence base, despite its potential benefits for increased fat metabolism (Cook et al., 2026), which could benefit longer‐duration endurance performance. Further research with larger sample sizes is required to determine the efficacy of blackcurrant supplementation on exercise performance.

### 2.9 Curcumin

#### 2.9.1 Effects on exercise recovery

Curcumin is the predominant (poly)phenol in the yellow rhizomes of turmeric spice and has been studied extensively for its anti‐inflammatory and antioxidant effects (Thorley et al., 2024). Interest in the use of curcumin as a recovery aid continues to grow, as evidenced by several recent systematic reviews and meta‐analyses examining their effects on markers of EIMD (Fang & Nasir, 2021; Oxley & Peart, 2024). The principal messages from these reviews, which contained 9 and 11 individual studies, respectively, were that although curcumin supplementation showed promise in attenuating delayed onset muscle soreness (DOMS) and creatine kinase (CK) concentrations, there was little evidence to support its benefits on the recovery of neuromuscular performance. In addition, there was limited evidence that curcumin impacts markers of inflammation and oxidative stress following exercise, partly because few studies (fewer than five) included these as measures, meaning that the potential mechanisms of action were unclear. The optimal dose is also not known, but one meta‐analysis (Fang & Nasir, 2021) suggested that doses of >180 mg/day were required to reduce postexercise CK levels markedly, because of the low bioavailability of (poly)phenols following ingestion (Thorley et al., 2024). It is important to note that although CK provides good evidence that muscle damage has occurred, it should not be used to provide forensic insight into the magnitude of damage. One recent addition to the literature showed that 1 week of supplementation with 1500 mg of hydrolysed curcumin reduced DOMS, CK and interleukin‐6 following damaging resistance training, but somewhat paradoxically, impaired recovery of performance in comparison to a lower dose of 750 mg and placebo control (Helder et al., 2025). The effects on DOMS after damaging eccentric exercise that are reported in several studies are promising, and these warrant further investigation in more rigorously designed studies that could provide some mechanistic insights (e.g., Nrf2 and downstream targets, prostaglandins).

#### 2.9.2 Effect on exercise performance

In contrast, studies examining the effects of curcumin supplementation on exercise performance are largely confined to animal models. In one example, a study in mice found that curcumin (50 mg/kg/day for 8 weeks) was able to increase treadmill running capacity, seemingly by augmenting Nrf2 activity in skeletal muscle (Wafi et al., 2019). In a recent human study, there was no effect of consuming 180 mg of curcumin 1 h before a soccer match on distance covered, high‐speed running distance or number of sprints and overall speed over the 90 min (Tanabe et al., 2024). Taken together, the studies conducted to date with curcumin suggest that supplementation might positively influence some aspects of EIMD, notably DOMS, but its effects on exercise performance in humans remain largely unexplored.

## 3 CONCLUSION

**FIGURE 2:**
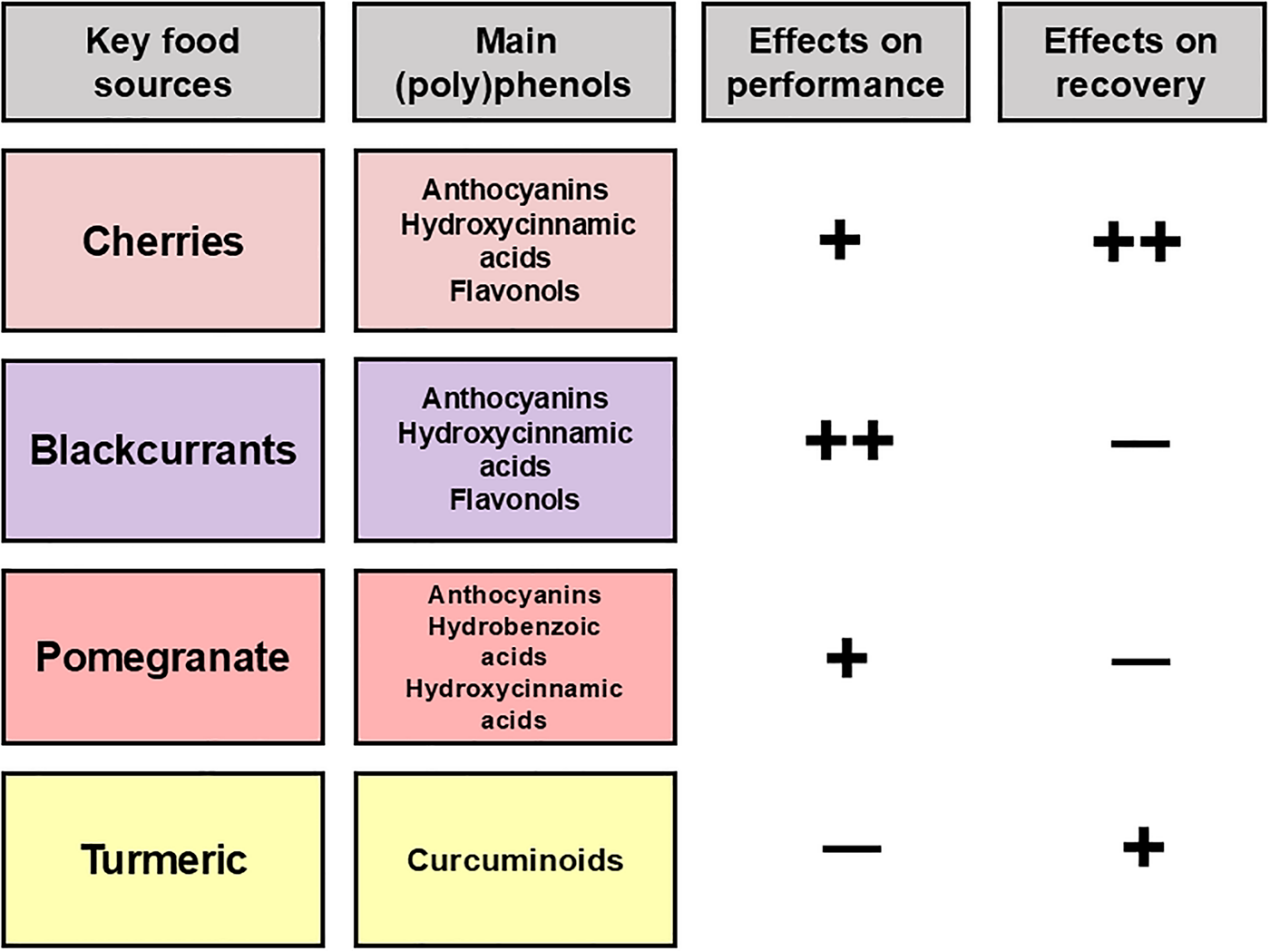
A schematic diagram of contemporary food sources that summaries the principal (poly)phenolic groups and the effects on performance and recovery. Key: —, currently inadequate evidence of beneficial effect owing to limited or equivocal evidence; +, effects are equivocal, but some evidence of beneficial effect; ++, strongest evidence base to support use.

There is compelling evidence from in vitro and animal studies that (poly)phenols can exert a range of biological effects that could impact exercise performance and recovery (for a summary, see Figure 1). Importantly, there is no evidence to suggest that (poly)phenol consumption impairs performance or recovery. At the very least, (poly)phenol‐rich foods contain essential nutrients and, in favourable conditions, have the potential to confer some ergogenic benefit. Based on our reading of the literature, in Figure 2 we have summarised the level of evidence associated with the exemplar (poly)phenol sources we focused on in this review. Although for many (poly)phenol supplements the data are largely equivocal, there is presently the strongest evidence of ergogenic performance benefits with blackcurrants and recovery benefits with cherries. Notwithstanding, most studies with these (poly)phenols and others examining the effects of (poly)phenols on exercise performance and recovery, at least in humans, have not quantified the plasma bioavailability of the (poly)phenols, which makes it difficult to determine causal effects, in addition to the dose required to elevate blood concentrations. The ability for humans to absorb and accumulate (poly)phenols in various tissues warrants further research, as does the need for a pre‐dosing strategy in human performance and recovery. This is an important step to provide evidence of causal mechanistic effects in well‐controlled clinical trials. Despite the lack of mechanistic data in humans, there is growing evidence that some (poly)phenol interventions can positively influence markers of recovery and performance, but the data remain inconsistent. The equivocal results to date are attributable, at least in part, to the wide range of different dosages and exercise protocols used, but also the low sample sizes in relatively heterogeneous populations. Despite many studies carrying out power calculations, an overarching criticism of the exercise science and sport nutrition literature is the modest sample sizes used, which could lead to erroneous conclusions and false positives or false negatives. In addition, both age and sex could affect performance and especially EIMD, with studies suggesting that older adults experience more, and females might experience less, symptoms of muscle damage (e.g., muscle soreness) than their younger or male counterparts (Li et al., 2024); however, the data are ambiguous, especially in females (Sayers & Clarkson, 2001). As such, the efficacy of (poly)phenols could be mediated, in part, by the age and sex of the individual. For example, given that older adults are more prone to oxidative stress and aberrant inflammation (Bouzid et al., 2014; Peake et al., 2010), it would be reasonable to assume that boosting antioxidant and anti‐inflammatory defences with (poly)phenols would confer greater benefits in this population than in younger adults, who, in the absence of disease, have more resilient antioxidant and immune defence systems. However, no studies to date have examined in detail how age or sex might mediate the effectiveness of (poly)phenols on these outcomes. A greater understanding of how factors such as these affect (poly)phenol efficacy, alongside a greater understanding of the primary mechanisms of action in humans, will enable more targeted and effective (poly)phenol‐based interventions for human performance and recovery.

## AUTHOR CONTRIBUTIONS

Glyn Howatson and Tom Clifford conceived the review, conducted the literature analysis, wrote the manuscript and approved the final version and agree to be accountable for all aspects of the work in ensuring that questions related to the accuracy or integrity of any part of the work are appropriately investigated and resolved. Both persons designated as authors qualify for authorship, and all those who qualify for authorship are listed.

## CONFLICT OF INTEREST

The authors declare no conflicts of interest.

## FUNDING INFORMATION

None.
